# Micro Milled Microfluidic Photoionization Detector for Volatile Organic Compounds

**DOI:** 10.3390/mi10040228

**Published:** 2019-03-30

**Authors:** Gustavo C. Rezende, Stéphane Le Calvé, Jürgen J. Brandner, David Newport

**Affiliations:** 1 Bernal Institute, School of Engineering, University of Limerick, V94 T9PX Limerick, Ireland; gustavo.coelho@ul.ie; 2 Université de Strasbourg, Centre national de la recherche scientifique (CNRS), ICPEES UMR 7515, F-67087 Strasbourg, France; slecalve@unistra.fr; 3 In’Air Solutions, 25 rue Becquerel, 67087 Strasbourg, France; 4 Institute of Microstructure Technology (IMT), Karlsruhe Institute of Technology, Hermann-von-Helmholtz-Platz 1, 76344 Eggenstein-Leopoldshafen, Germany; juergen.brandner@kit.edu

**Keywords:** photoionization detector, microfluidics, microfabrication, volatile organic compound (VOC) detection, toluene

## Abstract

Government regulations and environmental conditions are pushing the development of improved miniaturized gas analyzers for volatile organic compounds. One of the many detectors used for gas analysis is the photoionization detector (PID). This paper presents the design and characterization of a microfluidic photoionization detector (or µPID) fabricated using micro milling and electrical discharge machining techniques. This device has no glue and facilitates easy replacement of components. Two materials and fabrication techniques are proposed to produce a layer on the electrodes to protect from ultraviolet (UV) light and possible signal noise generation. Three different microchannels are tested experimentally and their results are compared. The channel with highest electrode area (31.17 mm²) and higher volume (6.47 µL) produces the highest raw signal and the corresponding estimated detection limit is 0.6 ppm for toluene without any amplification unit.

## 1. Introduction

Volatile organic compounds (VOCs) are a class of carbon-containing chemicals with a high vapor pressure at ambient temperature. Typical indoor sources of VOCs are varnishes, paints, solvents, cleaning materials, etc. In addition, outdoor sources, such as automobile and industrial waste, also contribute to both indoor and outdoor VOC pollution [1]. Many VOCs are harmful to humans, including benzene, which is carcinogenic and has no safe recommended level of exposure [2,3]. The European Commission established, with effect from 2010, a regulation for benzene exposure at a maximum limit of 5 µg/m³ (1.6 ppb) [4]. In addition, within the European Union, regulations and guidelines are established to maintain a healthy indoor air quality [4,5].

Air must be monitored in order to identify and quantify pollutants so that appropriate action can be taken to clean the air. Gas chromatographs (GCs) equipped with detectors are suitable gas analyzers to accomplish this task because they can separate, identify and quantify different chemicals, including VOCs. GCs in the market with low detection limits are still heavy, bulky, slow and lab-based [6,7,8], while current regulations raise the demand for efficient gas analyzers with enhanced portability, reduced resource consumption, improved robustness, high analysis speed, low cost and reduced detection limits. One possible way to achieve these improvements is through miniaturization of all the gas analyzer components, including the detector itself.

Among the existing detectors and techniques to detect VOCs [9], the photoionization detector (PID) is commonly used with a GC and it is suitable for miniaturization [10]. The PID uses ionization of gaseous compounds by light as a working principle to quantify chemicals in the gas samples. This detector can be classified according to the ionization source. When the ionization source is unseparated from the ionization chamber by a window, the ionization source is usually a discharge in a noble gas, such as helium, and is referred to as a discharge photo ionization detector (D-PID); or when the ionization source has no fluidic connection to the ionization chamber, which is known as the lamp photo ionization detector (L-PID). In both devices, a gas sample containing the species to be detected flows through the ionization chamber, where photons emitted by the ionization source reach the sample molecules. As a general rule, if the ionization energy of the photon is greater than the ionization potential of the molecule, ionization occurs. The electrodes establish an electric field in the ionization chamber where the ionized molecules generate an ionization current proportional to their concentration. Based on an external calibration, the electrical signal can be expressed as a chemical compound concentration.

Commercial detectors either have large ionization chamber volumes around 50–100 µL [11,12] or membranes, which increase the ionization chamber fill time [8,13]. For photoionization detectors applied to GC, reducing the external size of the PID is not the main point to ensure high efficiency, because existing portable PIDs are already small (20 mm) and lightweight (8 g) [8,13]. The challenge, therefore, is to develop a micro PID (µPID) detector with a rapid ionization chamber fill time, low gas flow rate and high sensitivity that is compatible for the development of a portable µGC equipped with a small carrier gas cylinder. This can be achieved with a miniaturized flow-through PID ionization chamber.

Reducing the ionization chamber volume of the PID can play an important role in overall GC-PID miniaturization. A small ionization chamber can improve both the gas analyzer performance and the portability. Ionization chamber miniaturization results in a higher surface to volume ratio, which should translate into a more sensitive signal. A small ionization chamber should also result in a higher signal-to-noise ratio. In addition to that, the smaller the ionization chamber volume is, the smaller the gas sample volume can be (without depleting signal intensity), and for a fixed carrier gas flow rate, this means also a faster analysis time and lower consumption of the carrier gas. Low carrier gas consumption is important to enable reduction in carrier gas cylinder size (reducing gas analyzer weight) and maintain carrier gas cylinder autonomy, both important qualities for portable GC-PID.

Recent scientific publications reveal innovations in the miniaturization of photoionization detectors, also in reducing the size of the ionization chamber. Table 1 shows the main publications, the type of PID, materials, design features and ionization chamber volume. These improvements in the ionization chamber volume were made possible by using silicon or etching based microfabrication techniques. Such techniques are often complex and expensive. This work proposes a new µPID prototype that uses simpler fabrication techniques, allows easy mount-dismount of the device components, easy exchange of parts and small ionization chamber volume.

## 2. Micro PID

This section describes the µPID design fabricated by micro milling and electrical discharge machining. The components are simply assembled together, dispensing any chemical or physical bonding process. Initially, the design, main components, fabrication and assembly are presented (divided in two sections: Core and shell), followed by a description of the electrode coating.

### 2.1. Design, Fabrication and Assembly

The detector has two main structures: The core, which is the heart of the device, responsible for the detection; and the shell, whose main functions are to clamp together all parts and enable the world-to-chip connections (electronic and fluidic). 

#### 2.1.1. Core

**Figure 1:**
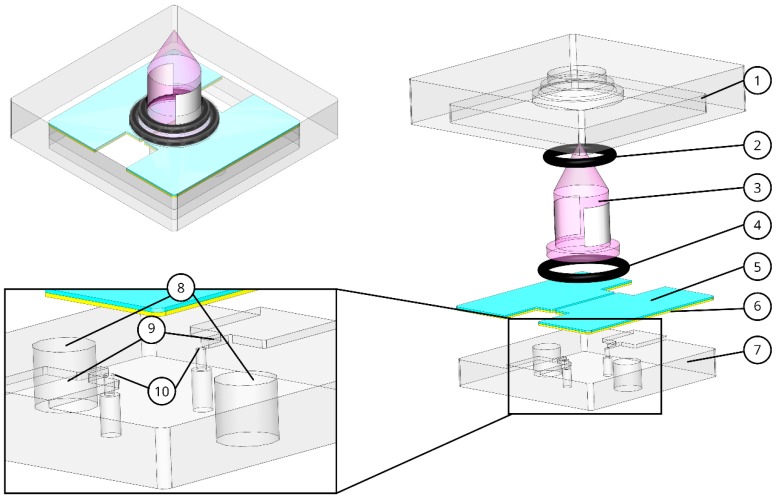
Assembled and exploded view of the detector core. (**1**) Top poly(methyl methacrylate) (PMMA), (**2**) O-ring 1, (**3**) UV lamp, (**4**) O-ring 2, (**5**) shield, (**6**) copper electrodes, (**7**) bottom PMMA, (**8**) electrode connection holes, (**9**) elevation structure, (**10**) micro-pores.

The components of the core when assembled yield the microchannel where the gas sample flows, which is the ionization chamber, where the detection of the sample occurs. The main parts of the core are numbered in Figure 1. The top and bottom poly(methyl methacrylate) (PMMA) are made using micro milling and the electrodes are fabricated with electrical discharge machining (both processes with tolerances ±0.02 mm). PMMA was chosen due to its transparency (to facilitate visual inspection of the assembly), its structural stability, chemical inertness to the VOC gas samples used and its low porosity (minimal sorption-desorption of the gasses passing through the ionization chamber). Copper was chosen for the electrodes owing to its high electrical conductivity and ready availability. The O-rings are made of Viton (ERIKS, Utrecht, The Netherlands), since its softness is required to avoid breaking the UV window which is fragile material (MgF_2_). In addition, a commercial UV lamp (Baseline MOCON, Lyons, CO, USA) with 10.6 eV output was used.

The bottom layer of PMMA constitutes the bottom wall of the microchannel, which contains two micro-pores of 500 µm diameter to form the inlet/outlet of the channel. It also has two holes to fit the electronic connectors to the copper electrodes. The electrodes fit on top of the bottom PMMA to form the lateral wall of the microchannel. The elevation in the bottom PMMA serves both to fit the electrodes to the right position in order to constitute the channel and to electrically isolate the two sides of the electrodes. To enclose the microchannel (ionization chamber), the UV lamp is placed on top of the copper electrodes, two O-rings with 1 mm cross section fit in the pockets designed on the top PMMA, which closes the channel and ensures that the lamp is fitted to the right position in the center of the fluidic path between the inlet/outlet pores. The top layer of PMMA is designed to fit tightly to the bottom PMMA layer to ensure alignment of the components and reduce the probability of leaks.

#### 2.1.2. Shell

**Figure 2:**
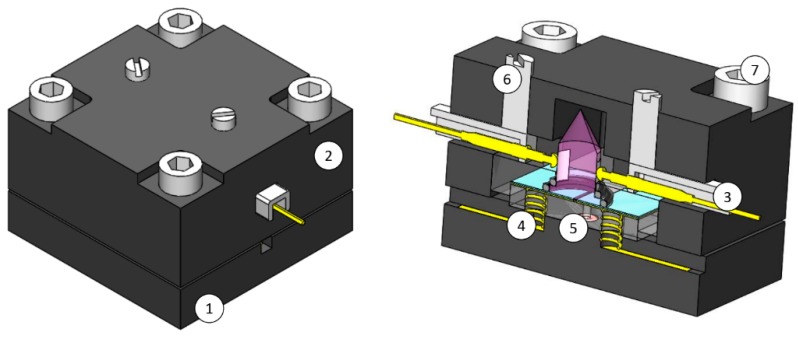
Isometric and cut view of the shell. (**1**) Bottom shell, (**2**) top shell, (**3**) lateral pin, (**4**) electrodes connection, (**5**) fluidic connections, (**6**) M3 pin fix screws, (**7**) M3 screw.

Another important structure is the shell of the device, which mechanically presses all the components together to minimize leakage and facilitates world-to-chip connectivity (fluidic and electronic). The electronic connections are to power the UV lamp, apply voltage on the electrodes and acquire the photoionization current. The fluidic connections connect the sample inlet and sample outlet. The main components of the shell are indicated in Figure 2. The top and bottom shells were made of PVC and fabricated using micro milling. The bottom shell has a groove to fit the spring and copper tape, which makes the electronic connection to the copper electrodes. A wire is soldered to the copper tape to connect the power supply and photoionization current signal acquisition. The bottom shell also has O-ring pockets (O-rings dimensions are: 3 mm outside diameter, 1 mm cross section and 1 mm internal diameter) on its top surface to connect the inlet/outlet gas sample to the ionization chamber. The O-rings are also made of Viton. The bottom surface of the bottom shell contains M3 threads to assemble the FESTO connector type QSM-M3-2 (Festo AG & Co. KG, Esslingen am Neckar, Germany), which is a plug in connector to 2 mm outside diameter tubing.

The top shell fits the top PMMA and positions the core so that it is aligned with the fluidic connections. It also encloses the UV lamp for safety measures. Two lateral pins are designed to slide freely and fit an electronic spring pin that reaches the lateral electrodes of the UV lamp. The spring allows freedom of the pin movement after touching the lamp, which is important to avoid impact and load being transferred to the fragile glass lamp. When the pins reach the right position, two M3 screws on top of the pins aid in fixing the pin to the right position. The lateral pins also ensure complete enclosing of the UV lamp to avoid its light. The top and bottom shells have passing holes for M3 screws that press all the parts at appropriate pressure to ensure fluidic sealing and structural stability, meaning no part would move unnecessarily during operation of the device.

Using no bonding might give rise to undesirable leakage. However, in comparison with other µPID designs [15,18,19,20,21,22], the device presented here has the following advantages: (1) Easy prototyping, since the fabrication techniques are more straightforward than other approaches adopted in the literature; (2) Lower fabrication cost, this device does not require clean-room complex and high-cost facilities and processes. In addition, the materials used are cheaper compared to silicon wafers and it is easy to exchange different microchannel geometries, if needed; (3) Easy mount-dismount, which facilitates maintenance and ease of substitution of components such as the UV lamp (e.g., to use lamps with different energies).

### 2.2. Ionization Chamber

The ionization chamber of the µPID is the microchannel formed between the bottom PMMA, top PMMA, lamp and electrodes. This design proposed can produce an ionization chamber with volume up to 100 times lower compared to some commercial PIDs. The height of the channel, 500 µm, is the thickness of the copper plate and the width is initially chosen to keep the aspect ratio of the microchannel close to 1, since a wider channel could increase unnecessarily the chamber volume and a smaller width could reduce excessively the UV illumination area. The length and shape of the microchannel are designed to maximize the use of the illumination diameter of the commercial UV lamp (estimated illumination diameter ~6 mm).

**Figure 3:**
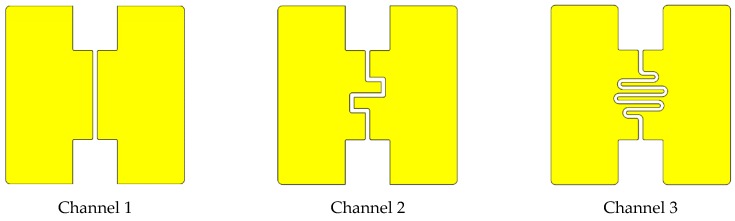
Microchannels for ionization chamber.

Important channel properties are: (1) Width, representing the distance between the copper electrodes; (2) Height, directly proportional to the electrode area; (3) Electrode area, which depends on the shape and the height of the microchannel; (4) Lamp illumination area, surface of the channel illuminated by the UV lamp and (5) Volume of the channel from inlet to outlet, which corresponds to the ionization chamber volume. Figure 3 shows three different electrodes/channel designs and Table 2 presents their dimensions. The three shapes displayed have the objective to change gradually the area of the electrodes and ionization chamber volume.

### 2.3. Coating Shield

When the copper electrodes are exposed to radiation at a frequency higher than the copper work function, electrons are ejected from the surface and cause signal noise. Therefore, it is desirable to protect the electrodes from the high-energy radiation coming from the UV lamp. A possible solution is to create an UV light coating shield on the copper surface. Ideally, a good shield will filter out all the radiation with an energy greater than the work function of the metal used as electrodes (in the case of copper, 4.48–4.94 eV [23]). In addition to that, it should be thin to ensure that the thickness of the coating does not increase the volume of the ionization chamber. Moreover, it must not cover the area of the electrode used for ion and electron collection. Other important characteristics are: Chemical compatibility with the electrode materials and gas samples, resistance to temperature oscillation, humidity and mechanical deformation. Two materials were considered in this paper for use as an electrode shield: Diamond-like carbon (DLC) and polymethyl methacrylate (PMMA).

#### 2.3.1. DLC Coating

Diamond-like carbon is a metastable form of amorphous carbon that can be used as a protective coating. It can be applied as thin film with dimensions ranging from a few nm to µm [24]. Even quite thin films can block unwanted radiation, which renders it potentially suitable as an electrode coating in this application. A 0.1 µm thickness layer of DLC can have 5–18% UV transparency at 350–450 nm wavelength range and 0.2 µm DLC thickness yields 2–8 % UV transparency [24]. Also, DLC preferentially blocks short wavelengths [25,26].

**Figure 4:**
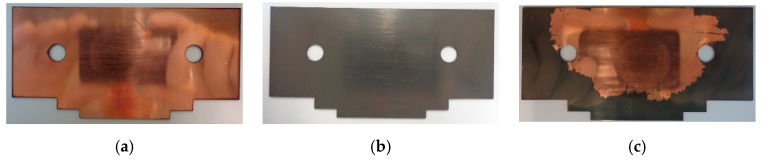
Diamond-like carbon (DLC) coating before and after failure. (**a**) Uncoated side before failure, (**b**) Coated side before failure, (**c**) Coated side after failure (4.5 weeks).

**Figure 5:**
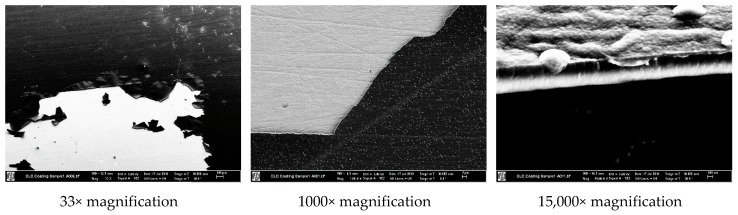
Scanning electron microscopy (SEM) images of the DLC coating failure.

A first sample of DLC coating was produced on one side of a 1 mm thick copper plate. Before the coating was applied, the surfaces were polished and etched (to remove impurities) for better adhesion of the DLC. The coating was made using plasma enhanced chemical vapor deposition and had 1 µm thickness. Figure 4 shows the difference between the coated and uncoated side. It can be seen that the DLC coating yields a grey surface on top of the copper. This sample started to peel off after a few weeks, likely due to exposure to sunlight and summer temperatures/humidity. Figure 4c shows the state of the peeled of sample. The scanning electron microscopy (SEM) images on Figure 5 shows that the DLC coating detached from copper like a peeled off thin foil, suggesting poor adhesion between the coating and substrate. A second sample was produced on the same type of substrate, this time incorporating a titanium adhesive layer between the DLC and copper to increase adhesion. The new sample had considerably better stability and did not fail (crack or break) during the test period.

#### 2.3.2. PMMA Coating

The second type of electrode coating considered is PMMA because it does not transmit wavelengths below 360 nm [27], which should be sufficient to avoid photoelectric effects in the copper electrodes. PMMA is a relatively cheap material compared to DLC and can be deposited as a top layer on the electrodes by spin coating, a process that is not expensive and does not require complex machinery. Disadvantages to be considered are: (1) Difficulty to produce perfectly flat surfaces to avoid leakage; (2) difficulty to produce very thin layers (< 0.1 µm) and (3) during fabrication, the PMMA might run inside of the channel, isolating the electrodes from the ions, then reducing the active area inducing a lower signal.

## 3. Results

Toluene was chosen as the representative compound of the VOCs for all the following experiments aiming at characterizing the µPID signal where different parameters were changed, such as voltage applied to the electrodes or total gas flow rate.

### 3.1. Experimental Setup and Material

**Figure 6:**
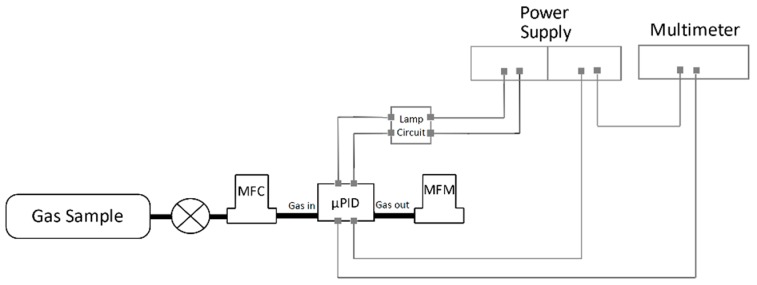
Experimental setup used for signal measurements from µPID prototype.

An experimental setup was built to evaluate the µPID prototype performance, represented in Figure 6. A gas sample supply, containing either pure nitrogen or toluene 100 ppm was connected to a mass flow controller (MFC), Bronkhorst El Flow Select F-201CV-050-AAD-33-V (Bronkhorst High-Tech BV., AK Ruurlo, The Netherlands), with a full scale of 20 mL/min and error of 0.5% of reading plus 0.1% of full scale. The MFC regulates the flow rate of gas sample into the µPID, and the flow rate leaving the device is read by a mass flow meter (MFM), Bronkhorst El Flow Select F-100D-AAD-33-V, full scale 20 mL/min and same error as the MFC. To power up the UV lamp, the lateral electrodes of the lamp are plugged inside the lamp drive circuit of the commercial Baseline MOCON PID. This circuit is powered by a power supply at 4V and yields a current of about 35 mA. The power supply was also used to provide the voltage on the electrodes in the ionization chamber and the digital electrometer Keithley 616A (Keithley Instruments, Solon, OH, USA) was used to measure the current signal.

### 3.2. Optical Microscope Images

**Figure 7:**
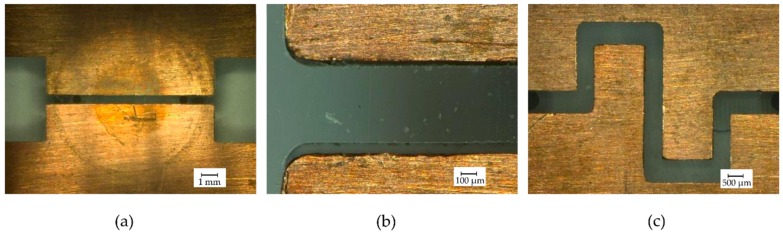
Digital optical microscope images of copper electrodes assembled on bottom polymethyl methacrylate (PMMA). (**a**) Channel 1; (**b**) Zoom at bottom PMMA and copper electrodes assembly; (**c**) Channel 2.

In order to investigate the effects of the fabrication and design, digital optical microscope images were taken from the microchannel formed by the electrodes and the bottom PMMA. Figure 7a shows the image of channel 1 previously used in experiments with the UV lamp. It is possible to notice round circles marked by the use of the lamp, the central circle suggest that UV light is more intense within a diameter of approximately 4.5 mm. The image zoomed on the fitting of PMMA and electrodes (Figure 7b) reveal imperfections of the fitting between them, which can lead to gaps ranging from 15–60 µm. The consequence of those gaps will be leakage and an undesirable variation in the distance between the electrodes. The fitting of electrodes presented in Figure 7a presented an average channel width of 504 µm (5.5% error), while the channel 2 shown in Figure 7c presented considerable deviations in the nominal value of channel width, varying from 454 µm to 548 µm.

### 3.3. Experimental Results and Discussion

The signals from the µPID were obtained without an amplifier, which should be developed in the future in order to improve the signal/noise ratio and thus the sensitivity. The multimeter (Keithley 616A) acquired µPID signals ranging from about 70 pA to 3.5 nA. The current standard uncertainty is calculated considering two main factors: (i) Multimeter resolution (R), which varies from 0.1 pA to 10 pA depending on the current measurement range and (ii) measurement repeatability, thus the combined standard uncertainty is given by

(1) $$u_{C}^{2}=u_{1}^{2}+u_{2}^{2}$$

The standard uncertainty calculated due to resolution and repeatability are obtained with

(2) $$u_{1}=R/\sqrt{3}$$

and

(3) $$u_{2}=\sigma \left(X\right)/\sqrt{n}$$

where σ(X) is the standard deviation of the sample and n are the number of samples. The expanded uncertainty (U) is calculated by

(4) $$U=t\cdot u_{C}$$

where t is the Student’s distribution factor for 95% confidence interval with v degrees of freedom calculated by the Welch-Satterthwaite Equation.

**Figure 8:**
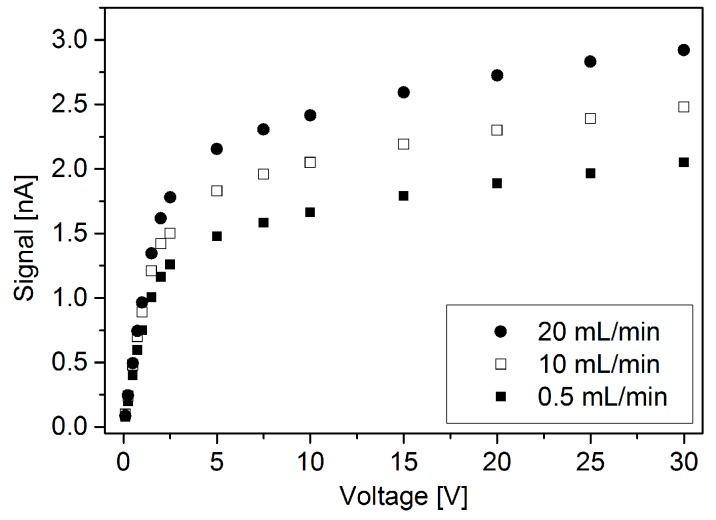
Channel 3 signal with variation of voltage on copper electrodes for various gas sample flow-rates. Uncertainty bars are not displayed in the graphic since their size is small compared to the symbols representing the points.

Figure 8 shows the µPID signal for channel 3 using toluene 100 ppm gas sample when voltage and flow rate are changed (leakage as a function of flow rate is presented in the supplementary file Figure S1). A range of voltages was applied from 0.1 V to 30 V and flow rates of 0.5, 10 and 20 mL/min were used. To obtain the data corresponding to the three flow rates, the µPID signal was stabilized, which lasted about 2h for 0.5 mL/min and 1h for 10 mL/min and 20 mL/min, this time being necessary to remove humidity traces which may perturb the signal. After stabilization, the voltage was varied from 0.1 to 30 V for each flow rate. Each point displayed on Figure 8 corresponds to an average of 10 signal measurements taken within 2 min interval at each flow rate and voltage. The repeatability is calculated individually for each point and corresponds to the standard deviation of the 10 measurements. The relative uncertainty of the µPID signal (U/Signal) varies from 7.5% to 0.1% at low and high voltages respectively.

The three flow rate datasets (0.5, 10 and 20 mL/min) present similar curve shape and the signal increases with flow rate. Two linear regions of the data can be identified: (i) 0.1 V to 2.5 V and (ii) 5V to 30 V. The slope of the first region is ~ 25 higher than the second region. At 5 V, signals for 0.5, 10 and 20 mL/min are about 1.5, 1.8 and 2.2 nA, meaning that low flow rates might not be so detrimental for signal generation.

**Figure 9:**
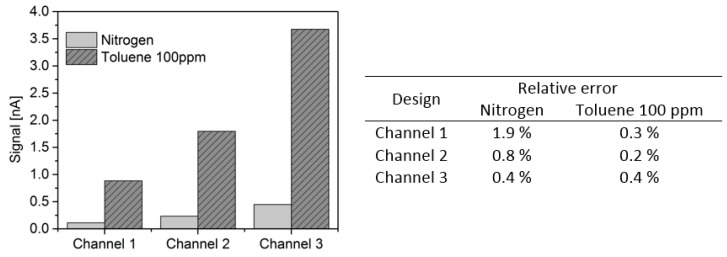
Signal for Channels 1, 2 and 3 using nitrogen and toluene 100 ppm as gas samples. Flow rate 20 mL/min and 30 V on electrodes. Relative uncertainty for signals of Channel 1 with nitrogen and toluene 100 ppm are 1.9% and 0.3%. Relative uncertainty for signals of Channel 2 with nitrogen and toluene 100 ppm are 0.8% and 0.2% respectively. Relative uncertainty for signals of Channel 3 with nitrogen and toluene 100 ppm are both 0.4%.

**Figure 10:**
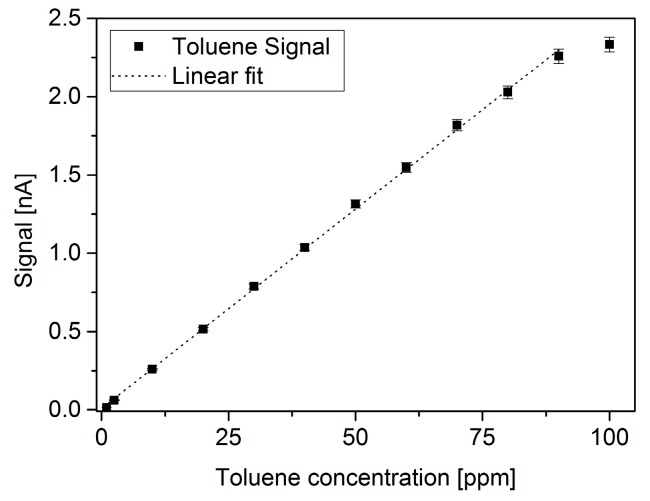
Channel 3 signal at 1–100 ppm toluene range. 20 mL/min and 30 V applied. Relative error is 2% for all data points.

For gas analyzer portability, low flow rate and low voltage are desirable specifications, which means longer duration of portable carrier gas cylinder and longer battery life. As an initial analysis from Figure 8, a voltage of 5 V and flow rate 2 mL/min, might be a good choice to yield best cost benefit of portability vs. µPID signal level. However, since the currents generated by the device are very low, and those experiments were performed without a signal amplifier, the flow rate and voltages yielding the highest signal possible were chosen for the rest of the work and in particularly to obtain the results shown in Figure 9 and Figure 10.

In order to compare the performance of the three channels fabricated, Figure 9 presents the current signal for nitrogen and toluene 100 ppm for each of the three channels. The gas sample was injected at 20 mL/min and 30 V was applied on the electrodes. The current was measured after signal stabilization and five measurements were taken, the values displayed on Figure 9 are the average. The same procedure was adopted to calculate the uncertainties of this data. However, the repeatability corresponds to the average of the five measurements taken after stabilization. The values of uncertainties are given in the table of Figure 9 and the highest yielded value was 1.9%.

When comparing the signal for the three channels, it is evident that higher area yields higher signal. Also, the difference between the nitrogen and toluene 100 ppm signal increases, meaning higher sensitivity and possible better detection limit. Channels 1 and 2 have both 500 µm as nominal channel width, and it is interesting to notice that the proportion of area increase from channel 1 to channel 2 is similar to the signal increase observed, about 2 times higher. It is important to note that the signal of channel 3 for toluene 100 ppm was taken on a different day, yielding a level slightly higher than that observed in Figure 8.

Because the signal is higher with channel 3, the following measurements were performed in this configuration. Figure 10 shows signal of channel 3 for various toluene concentrations, ranging from 1 to 100 ppm. Again, the same conditions of voltage and flow were applied to get this data (30 V, 20 mL/min). Before and after measuring the signal of toluene, the µPID was purged with nitrogen. The data shown corresponds to the values of the signal for toluene at each concentration subtracted by the average of the purge signal (pure N_2_) obtained before and after the toluene signal acquisition. It can be noticed that up to 90 ppm, the response of the µPID is linear and a linear fit can be obtained (r² = 0.9996), which is presented in the graphic. The relative error is calculated with same procedure as in Figure 8 and Figure 9, yielding a maximum of error of 2%.

To estimate the detection limit, the maximum variation of the blank within a 2 min interval was obtained (after stabilizing) $\Delta S_{\max}$ = 0.005 nA. The detection limit for toluene is the concentration corresponding to $3\times \Delta S_{\max}$ (0.015 nA), which is estimated from a rule of three with the lowest concentration investigated, i.e., 2.5 ppm and 0.063 nA from the graph. The resulting estimated detection limit is therefore 0.6 ppm.

## 4. Conclusions

This paper presents the design, fabrication and characterization of micro milled µPID. The design’s simple construction allows easy mount-dismount for components change, cleanse or replacement. The ionization chamber volume ranges from 1.75 to 6.42 µL, depending on the electrodes. Two materials have been proposed for electrode shielding. DLC needs to have a titanium adhesive layer for improved stabilization on the surface of copper electrodes. PMMA structures on copper electrodes are stable. However, the fabrication technique should be improved for adhesion only on top surface of the electrodes.

Optical microscope images of microchannels shows that electrodes might not fit well on bottom PMMA, possibly leading to distortion in the width of the microchannel and moderate leakage. µPID signal level increases with flow rate, voltage and electrode area. Channel 3 has highest signal and electrode area. However, it has the highest ionization chamber volume. The device presents an estimated detection limit of 0.6 ppm for toluene. Future design improvements should be done to mitigate leakage, improve electrodes fitting on bottom PMMA. Future evaluations will be done on response time, residence time, possible adsorption of analytes on the interior surfaces, influence of pressure and humidity on the signal. The device will also be compared to commercial detectors and coupled to a separation column and pre-concentrator.

## Figures and Tables

**Table 1:** Main works on microfluidic photoionization detector (µPIDs).

Reference	Ionization Source	Manufacturing Main Materials	Design Main Features and Dimensions	Ionization Chamber
[14]	UV Lamp, 10.6 eV	-	Introduced nozzle inside a conventional ionization chamber.	10 µL
[15]	UV Lamp, 10.6 eV	Highly doped p-type <100> single-sided polished conductive Si wafers with resistivity 0.001–0.005 Ω.cm and 380 µm thickness; 500 μm thick Pyrex glass wafers.	Ionization chamber is a microchannel with cross-section 150 μm (width), 380 μm (depth) and length 2.3 cm. Entire overall channel size is 15 mm × 15 mm. Microchannel area covered by lamp is 2.4 mm × 2.4 mm.	1.3 μL
[16]	UV Lamp, 10.6 eV	Conductive p-type <100> silicon wafer and glass.	Channel etched 380 μm (width) × 380 μm (depth) × 2 cm (length).	0.5 µL
[17]	Helium discharge	Silicon and glass architecture.	Micro separation column fabricated on the same chip. Overall size (1.5 cm × 3 cm)	Not mentioned
[18]	Helium discharge	500 μm thick p-type <100> double side polished Si wafer with 500 nm thick thermal oxide layers; 100 μm thick Borofloat 33 glass wafer; 500 μm thick Borofloat 33 glass wafer.	Microchannels formed by Si and glass. Three main channels: 1) Auxiliary helium; 2) Analytes; 3) Outlet channel. Cross-section 380 μm (width) and 500 μm (depth);	1.4 µL
[19]	Helium discharge	Two (bottom and top) Borosilicate glass wafers 700 μm thickness and 100 mm diameter used as substrate.	Channel etched 250 µm (depth).	Not mentioned
This work	UV Lamp, 10.6 eV	Micromilled PMMA and PVC. Copper plate.	Modular assembly of components. No use of glue. Microchannels width vary between 400 and 500 µm.	1.75–6.42 µL

**Table 2:** Ionization chamber properties.

Channel *n*	w [µm]	A_electrodes_ [mm²]	V_inlet/outlet_ [µL]	A/A_1_	V/V_1_
1	500	5.98	1.75	1.0	1.0
2	500	12.67	2.75	2.1	1.6
3	400	31.17	6.42	5.2	3.7

## Supplementary Materials

The following is available online at https://www.mdpi.com/2072-666X/10/4/228/s1, Figure S1: Leakage for three channel designs. Uncertainty varies from 1% at high flow rates to 5% at low flow-rates.
