# Supplementary material for: Micro Milled Microfluidic Photoionization Detector for Volatile Organic Compounds
Source: Micromachines (Basel). 2019 Mar 30;10(4):228. doi: 10.3390/mi10040228 (PMC6523568; doi:10.3390/mi10040228)
Supplement: Supplementary file 1 [file micromachines-10-00228-s001.pdf]

Micro Milled Microfluidic Photoionization Detector for Volatile Organic Compounds

Gustavo C. Rezende, Stéphane Le Calvé, Jürgen J. Brandner and David Newport

Leakage for experiments with channel 1, channel 2 and channel 3 using nitrogen at different flow rates:

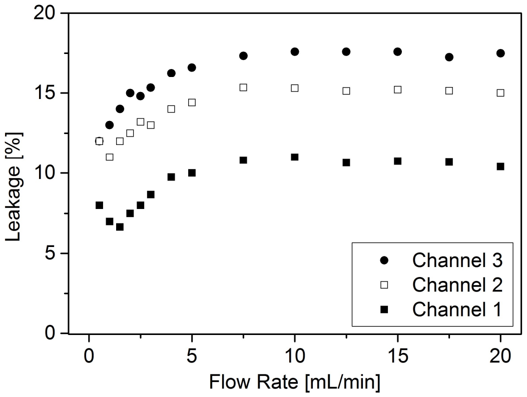

Figure S1. Leakage for three channel designs. Uncertainty varies from 1% at high flow rates to 5% at low flow-rates.

$$\text{Leakage [\%]} = \frac{\text{(Inlet flow rate)} - \text{(outlet flow rate)}}{\text{(Inlet flow rate)}} \times 100$$
